# Pancreatic Cancer and the Obesity Epidemic: A Narrative Review

**DOI:** 10.7759/cureus.26654

**Published:** 2022-07-08

**Authors:** Devyani S Poman, Lakshya Motwani, Nailah Asif, Apurva Patel, Deepanjali Vedantam

**Affiliations:** 1 Research, Smolensk State Medical University, Smolensk, RUS; 2 Research and Development, Smt. Nathiba Hargovandas Lakhmichand (NHL) Municipal Medical College, Ahmedabad, IND; 3 Research, Ras Al Khaimah (RAK) College of Medical Sciences, Ras Al Khaimah, ARE; 4 Research, Gujarat Medical Education & Research Society (GMERS) Medical College, Gotri, Vadodara, IND; 5 Internal Medicine, Kamineni Academy of Medical Sciences and Research Center, Hyderabad, IND

**Keywords:** chronic pancreatitis, diabetes mellitus type 2, type b insulin resistance, obesity-related illnesses, malignant pancreatic cancer

## Abstract

Pancreatic cancer (PC) is one of the most frequent causes of death. It usually affects older individuals with incidence closely approaching mortality due to its early asymptomatic feature and highly metastatic nature. Multiple risk factors such as family history, smoking, and germline mutations are associated with PC development, with obesity being one of the controllable factors. This review article focuses on the compilation of various studies to help establish a correlation between obesity or an increased body mass index and PC development. Hence, in this review, we have summarised multiple biological mechanisms of PC development induced by obesity, including insulin resistance, inflammation, beta-cell dysfunction, and oxidative stress, to prove that their correlation when combined with other factors, such as smoking, alcohol and chronic pancreatitis, may increase its risk. We have also reviewed potential diagnostic and screening techniques, such as evaluating precancerous lesions in high-risk patients and management plans discussing upcoming advances in treatment tactics such as neoadjuvant therapy, to reduce post-operative complications.

## Introduction and background

The first recorded description of pancreatic cancer (PC) is credited to Giovanni Battista Morgagni in his 1761 work *De Sedibus et Causis Morborum per Anatomen Indagatis* [1]. Then, Jacob Mendez Da Costa examined Morgagni's initial research and recorded the first microscopic diagnosis of adenocarcinoma in 1858, which was a significant step forward in our understanding of PC. PC is the 12th leading cause of cancer in the United States, with 50,000 individuals diagnosed yearly, with a five-year survival rate of newly diagnosed individuals (including men and women) at 8% [2]. Even though the mortality rate of PC has declined slowly through the years, there has been an inexplicably growing incidence rate [3-5]. Pancreatic ductal adenocarcinoma (PDAC) is expected to be the second biggest cause of cancer death by 2024, surpassing liver cancer in men and breast cancer in females [6,7]. This rising fatality rate associated with PC can result from people being diagnosed at advanced stages of the disease due to its asymptomatic presentation when it is consequently incurable. The 2017 Global Burden of Disease Study, a systematic attempt to portray the global burden of various diseases from 1990 to 2017 across 195 countries and territories, demonstrated a 2.3-fold rise in worldwide PC cases and deaths, with a three-fold higher incidence in countries with more significant social-demographic indices [8]. As a result, recognising potential risk factors for PC, implementing better screening measures, and enlightening the general public are critical steps toward minimising mortality [9,10]. PC occurs at a different incidence rate in other countries and populations. Northern America (7.4 per 100,000 people) and Western Europe (7.3 per 100,000 people) had the highest PC incidence rates in 2012, compared to Middle Africa and South-Central Asia, which had the lowest rates (about 1.0 per 100,000 people) [11,12].

Pancreatic ductal adenocarcinoma is the most common subtype of PC [5]. Genetics are a non-modifiable factor that accounts for about 10%-15% of pancreatic ductal adenocarcinomas. Many factors can cause PC, of which strong family history is the most significant. Some high-risk genes that have been linked to PC include BRCA1/2, PALB2, ATM, TP53, MLH1, STK11/LKB1, APC, CDKN2A, and SPINK1/PRSS1. Obesity, type 2 diabetes (T2D), and smoking are potentially modifiable variables for pancreatic tumour formation [13,14]. While smoking is the most prominent environmental risk factor for PC, epidemiological studies have linked obesity to a greater likelihood of developing PC [15,16]. A major National Institutes of Health cohort study found that individuals who were obese (defined as having a body mass index, or BMI, of 30 kg/m^2^) had a higher risk of acquiring this malignancy than patients with BMIs in the normal range, with hazard ratios (HRs) ranging from 115 to 153 kg/m^2^ [17]. Fatty infiltration of the pancreas has been correlated with the development of pancreatic intraepithelial neoplasia, a precursor of pancreatic ductal adenocarcinoma [18]. In a 2019 published study of cancer patterns among young adults in the United States, researchers discovered a dramatic increase in the incidence of obesity-related cancers, such as the increased risk of colorectal, endometrial, PCs and multiple myeloma, among patients aged 25-49 years [19]. The fast growth in overweight or obesity prevalence in the United States may have influenced these patterns. Obesity prevalence in the United States grew by more than 100% (from 14.7% to 33.4%) among children and adolescents between 1980 and 2014 and by 60% among adults aged 20-74 years (from 48.5% to 78.2%) [20]. This article aims to review how obesity or being overweight (increased BMI) and other related factors such as diet and physical activity may influence the development of pancreatic intraepithelial neoplasia and thus pancreatic neoplasm.

## Review

Epidemiology of obesity and PC

Obesity, combined with smoking, is among the few controllable risk factors that increase the risk of PC. According to monumental evidence, obesity-related diseases increase the incidence of PC, including pooled and meta-analyses (Table 1). A systematic review and meta-analysis conducted by Aune et al. in 2012 including 9504 patients from 23 prospective studies of BMI and PC risk found that both general and abdominal fatness increased PC even when the data were filtered by gender and geographic location, with a summary relative risk (RR) of 1.10 (95% confidence interval, or CI, 1.07-1.14, I^2^ = 19%) [21]. Similarly, Arslan et al. employed data from the National Cancer Institute's (NCI) Pancreatic Cancer Cohort Consortium (PanScan) in a case-control study with 2170 cases and 2209 control subjects [22]. They adjusted for confounders to investigate the link between pre-diagnostic anthropometric parameters such as BMI and PC risk. They found a positive correlation between BMI and the probability of developing PC (adjusted odds ratio [OR] for the highest vs. lowest BMI quartile = 1.33, 95% CI = 1.12-1.58, P_trend_ < 0.001). The impact of central adiposity on PC development was also investigated. They concluded that having a high waist-to-hip ratio was associated with a 20%-30% higher risk of PC [22]. Another study conducted by Pang et al. in China, published in 2017, recruited 512,891 adults aged 30-79 years from 2004 to 2008, recording 595 incident cases of PC during an eight-year follow-up [23]. They adjusted HRs for PC linked with self-reported young adulthood (mean 25 years) BMI and measured adulthood (mean 52 years) BMI and other adiposity parameters (e.g., waist circumference). They were further meta-analysed with published prospective studies. Overall, the mean BMI (SD) was 21.9 (2.6) kg/m^2^ at age 25 years and 23.7 (3.3) kg/m^2^ at age 52 years. There was a strong link between young adulthood BMI and PC (adjusted HR = 1.36; 95% CI 1.16-1.61, per 5 kg/m^2^ higher BMI). There was also a correlation between PC and adulthood BMI (adjusted HR = 1.11; 95% CI 0.97-1.27, per 5 kg/m^2^) [23]. Unlike the studies mentioned above, Lin et al. performed a hospital-based case-control study in Japan that consisted of genotyping rs9939609 in the fat mass and obesity-associated (FTO) gene of 360 cases and 400 control subjects that proved to be different [24]. The study investigated whether genetic variations in the FTO gene (rs9939609 allele) were associated with PC risk. After adjusting for sex, age, BMI, cigarette smoking, and history of diabetes, those with the heterozygous TA genotype and the minor homozygous AA genotype had a 48% (OR 1.48; 95% CI 1.07-2.04) and 66% (OR 1.66; 95% CI 0.70-3.90) increased risk of PC, respectively. The results indicated that the risk of PC was not linked to BMI. Instead, an association between a higher risk of PC in Japanese people and the FTO gene was found, presumably through a mechanism unrelated to obesity [24].

**Table 1:** Studies showing an association between an increased BMI and PC PC, pancreatic cancer; BMI, body mass index; FTO, fat mass and obesity-associated gene; WHR, waist-to-hip ratio

Author	Design	No. of cases	No. of controls	Population/location	Conclusion
Pang et al. (2017) [23]		595		512,891 adults aged 30-79 years	There was a strong link between young adulthood BMI and PC and also a correlation between PC and adulthood BMI.
Kasenda et al. (2014) [25]	Retrospective cohort study	483		Conducted in four Swiss hospitals between 1994 and 2004	In a multi-variable analysis, individuals who were obese and diagnosed with advanced PC had a worse prognosis than patients who were not obese.
Lin et al. (2013) [24]	Hospital-based case-control study	360	400	Conducted in Japan	Risk of PC was not linked to BMI. Instead, an association between a higher risk of PC in Japanese people and the FTO gene was found through a mechanism unrelated to obesity.
Aune et al. (2012) [21]	A systematic review and meta-analysis	9504			Both general and abdominal fatness increased PC even when the data were filtered by gender and geographic location.
Arslan et al. (2010) [22]	A case-control study	2,170	2,209	Pancreatic cancer cases and controls aged between 37 and 94 years	There was a positive correlation between BMI and WHR and the probability of developing PC.
Li et al. (2009) [26]	A case-control study	841	754	Conducted at a university cancer centre in the United States from 2004 to 2008, matched by age, race, and sex	Early-adulthood obesity or overweight was linked to a younger age of disease onset and a higher risk of PC. Patients with PC who were obese at an older age had worse overall survival rates.

A multicentre cohort study by Kasenda et al. in Switzerland used multivariable Cox regression to explore the effect of obesity on the prognosis of PC in 483 patients [25]. This study included patients with advanced or metastatic PC treated at four hospitals between 1994 and 2004. Patients were divided into four BMI groups: <18.5, 18.5-25, ≥25-29, and ≥30 kg/m^2^. The results indicated that in multivariable analysis, increasing BMI and obesity (HR 1.22; 95% CI 1.04-1.41, P = 0.012) were significantly associated with a worse survival prognosis [25]. Another case-control study that supported the link between PC and obesity independent of other factors was conducted by Li et al. in the United States from 2004 to 2008 [26]. It included 841 patients with pancreatic adenocarcinoma and 754 healthy individuals frequency-matched by age, race, and sex. Height and body weight histories were also recorded. Unconditional logistic, linear, and Cox proportional hazard regression models were used to investigate the relationships between patients' BMI and risk of PC, age at onset, and overall survival, respectively. Independent of diabetes status, individuals who were overweight (BMI 25-29.9; highest OR 1.67; 95% CI 1.20-2.34) or obese (BMI 30; highest OR 2.58; 95% CI 1.70-3.90) from the ages of 20 to 49 years had an elevated risk of PC. PC began two to six years earlier in people who were overweight or obese from the ages of 20 to 49 years. Individuals who were overweight or obese from the ages of 30 to 79 years had a lower overall survival of PC compared to those with normal weight and after adjusting for multiple clinical factors, irrespective of the stage of cancer and tumour resection status (overweight patients: HR 1.26; 95% CI 0.94-1.69, P = .04; obese patients: HR 1.86; 95% CI 1.35-2.56, P = .001) [26].

Pathogenesis

Obesity increases the risk of developing PC through a variety of mechanisms. One is how obesity contributes to diabetes mellitus (DM), a known risk factor for PC development.

Obesity, inflammation, and insulin resistance

**Figure 1:**
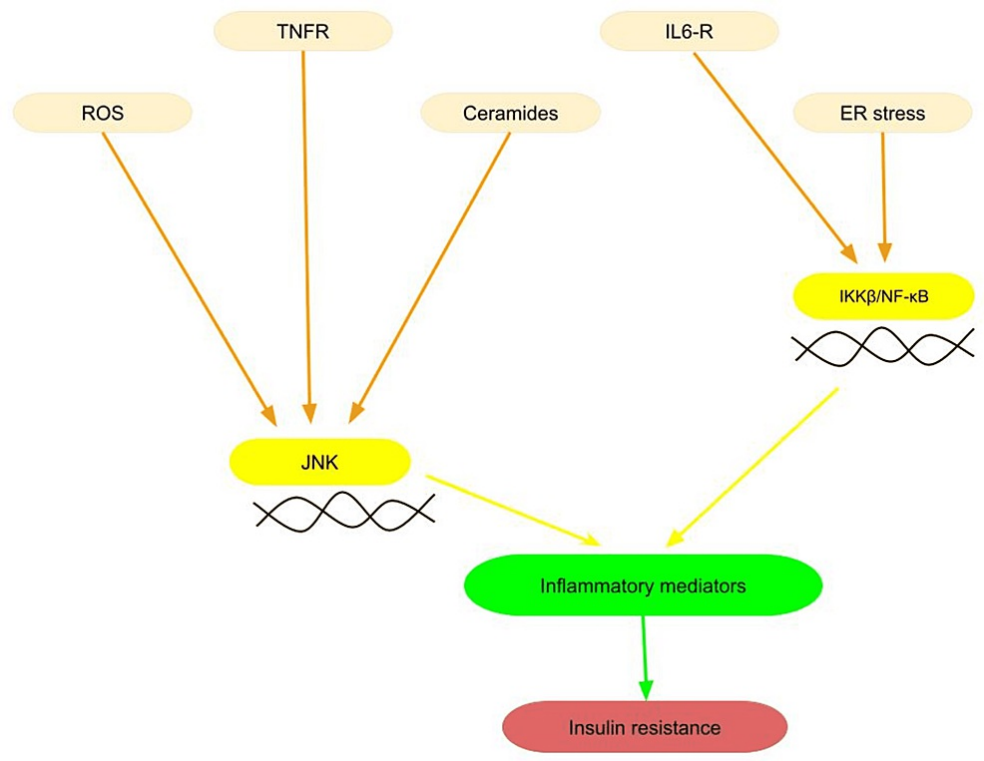
Inflammatory mediators and the pathways leading to insulin resistance ROS, reactive oxygen species; TNFR, tumor necrosis factor-receptor; IL6-R, interleukin 6-receptor; ER, endoplasmic reticulum; JNK, c-Jun N-terminal kinase; IKKβ/NF-κB, inhibitory-κB kinase/nuclear factor-κB

Adipose tissue influences metabolism by releasing non-esterified fatty acids (NEFAs), glycerol, and hormones like leptin, adiponectin, and proinflammatory cytokines. It also reveals an increase in the output of NEFAs, leptin, and cytokines such as tumour necrosis factor-alpha (TNF-α), interleukin-6 (IL-6), and monocyte chemoattractant protein-1 (MCP-1) and a decrease in adiponectin production. When triggered by pancreatic inflammation, latent pancreatic stellate cells (PSCs) become myofibroblast-like cells expressing alpha-smooth muscle actin (α-SMA) and numerous extracellular matrix (ECM) proteins, growth factors, and cytokines through anatomical and functional modifications [27]. The beginning of fibrogenesis is dependent on repeated and chronic pancreatic damage and inflammation. Activated PSCs are important in PC because they produce ECM proteins, regulate desmoplastic processes, and promote cancer cell proliferation, migration, and invasion. Furthermore, PSCs promote angiogenesis, required for tumour development and metastasis, resulting in tolerance to traditional chemotherapeutic agents and, consequently, treatment failure [28]. An increased production of TNF, IL-6, MCP-1, and other products of macrophages and other cells that occupy adipose tissue may consequently also play a role in the development of insulin resistance. TNF-α and IL-6 stimulate both the c-Jun N-terminal kinase (JNK) and the inhibitory-κB kinase (IKK)/nuclear factor-κB (NF-κB) pathways through conventional receptor-mediated mechanisms, resulting in overexpression of possible inflammatory mediators that can eventually contribute to insulin resistance (Figure 1) [29-33]. Adiposity also increases systemic indicators of oxidative stress, e.g., reactive oxygen species (ROS). This can be explained by the lipid build-up in the adipocyte that activates NADPH oxidase, increasing its generation. Other changes include increased cellular stresses, such as endoplasmic reticulum (ER) stress, which along with ROS, may activate JNK and NF-κB pathways [34,35]. Alternatively, oxidative stress might trigger a cascade of biological changes, comprising DNA damage, inflammation, apoptosis, and insulin resistance, contributing to cancer formation [36,37]. Ceramides can result from exposure to cell stress and increase cellular signalling, including apoptosis regulation. Ceramides, which build up in tissues like muscle and are associated with insulin resistance, may be increased by an intake of saturated fats. Ceramides also activate inflammatory pathways, including JNK and NF-κB, contributing to insulin resistance [38,39].

Obesity, beta-cell dysfunction, and insulin resistance

One of the prominent causes of type 2 DM is a persistent deterioration in beta-cell function. According to research, fasting and postprandial blood glucose levels rise when beta-cell malfunction leads to insufficient insulin production. The release of NEFAs may be the most significant factor in insulin sensitivity regulation. Humans acquire insulin resistance within hours after an acute elevation in plasma NEFA levels [40-43]. Increasing plasma NEFA levels in obesity may lead to the persistent loss of function of beta-cells, causing severe dysfunction in glucose-stimulated insulin secretion pathways and impaired insulin biosynthesis.

Insulin resistance, DM, and PC

**Figure 2:**
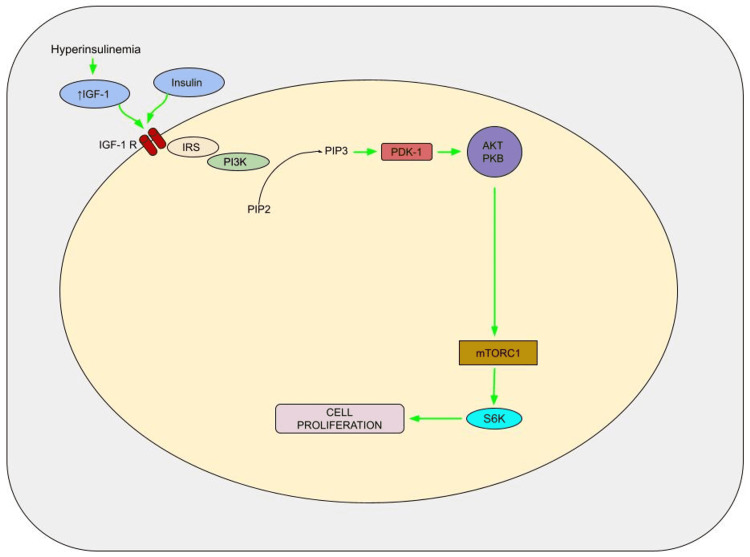
Pathogenesis of hyperinsulinemia-induced cell proliferation IGF-1, insulin-like growth factor-1; IGF-1 R, IGF-1 receptor; IRS, insulin receptor substrate; PI3K, phosphatidylinositol 3-kinase; PIP2, phosphatidylinositol 4,5-bisphosphate; PIP3, phosphatidylinositol 3,4,5-triphosphate; PDK-1, 3-phosphoinositide-dependent protein kinase 1; AKT/PKB, protein kinase B; mTORC1, mammalian target of rapamycin complex 1; S6K, p70 ribosomal S6 kinase

Peripheral insulin resistance and compensatory hyperinsulinemia are common findings in people with type 2 DM [44]. Insulin is a growth-promoting hormone that promotes cancer progression by boosting cell proliferation, reducing apoptosis, increasing glucose consumption, and influencing the concentrations of other factors contributing to tumorigenesis [45,46]. For example, reducing the formation of insulin-like growth factor (IGF)-binding proteins increases the quantity of accessible IGF-1 [47]. IGF-1 is a more powerful mitogen than insulin, promoting cell proliferation and invasion of PC cells (PCCs) while suppressing tumour suppressors such as phosphatase and tensin homolog. The PI3K/Akt and Raf/MAPK pathways can activate by either insulin or IGF-1 interacting with the IGF-1 receptor, promoting cell proliferation and inhibiting apoptosis (Figure 2) [44,48,49].

Other mechanisms of obesity leading to PC

Adiponectin is produced and released by fatty tissue and found in high concentrations in the blood (between 5 and 10 g/mL) [50]. Increased serum adiponectin levels reduce cancer risk, whereas low levels raise the risk [51]. Even though the molecular basis of adiponectin's anti-tumour effect is uncertain, it might have a role in developing PC through various possible methods. First, by tyrosine phosphorylation of insulin receptors in muscle tissue, adiponectin can directly enhance insulin sensitivity and reduce insulin resistance, resulting in decreased insulin/IGF-1 signalling [52]. Additionally, adiponectin is an anti-inflammatory cytokine that may block NF-κB activation and down-regulate the production of inflammatory cytokines like TNF-α and IL-6, which is believed to be the molecular mechanism behind these pathological conditions [53]. Decreased adiponectin concentrations will cause the generation of inflammatory cytokines in obese people, leading to the development of DM and, consequently, cancer. Adipocytes produce leptin, which is linked to obesity and insulin resistance [54]. Studies have indicated that leptin's anti-apoptotic and pro-angiogenic action is mediated via vascular endothelial growth factor (VEGF) activation, suggesting that it might be proinflammatory. Furthermore, when leptin binds to its receptor, it activates the PI3K/Akt/MAP kinase and STAT signalling pathways, essential for cell survival, growth, proliferation, and differentiation [54,55]. As a result, leptin may play a crucial role in the pathogenesis of PC.

A link has been found between DM and PC development as an independent risk factor (Table 2) [56]. A study by Aggarwal et al., published in 2013, focusing on both sexes and with a mean age of around 71.6, reviewed the prevalence of DM in 100 PC patients, 400 patients of other cancers and 100 noncancer controls [57]. The findings suggested a significantly (P < 0.0001) greater prevalence of DM (68%) in patients with PC compared to age-matched patients with other cancers. In another open study conducted in Sweden including 44 patients, Permert et al. investigated glucose tolerance and insulin secretion in PC patients compared to healthy controls [58]. They concluded that 75% had impaired glucose tolerance or diabetes. These studies, however, did not consider whether DM was a consequence of PC or a cause of PC. In a case-control study including 1621 pancreatic adenocarcinoma cases and 1719 matched controls from 12 cohorts, Elena et al. studied the association between DM and PC [59]. Race, sex, age, alcohol usage, smoking, BMI, and family history of PC were all adjusted, excluding patients with new-onset DM (less than two years). The study concluded that DM increased the risk of PC by 40% (OR 1.40; 95% CI 1.07-1.84). The risk was highest for those with a DM duration of two to eight years (OR 1.79; 95% CI 1.25-2.55); there was no connection for those with a DM duration of more than nine years (OR 1.02; 95% CI 0.68-1.52) [59]. Zhang et al. in a study in China investigated the risk of PC in DM patients [60]. They compiled 26 case-control studies involving 7702 PC cases and 10,186 controls, with the results indicating that the diabetes-PC association had an estimate of 3.69 (95% CI 3.12-4.37). Furthermore, the chance of acquiring PC was inversely related to the length of diabetes. Individuals with DM for less than two years had a higher than twofold increased risk of PC compared to those with DM for 2-4 or 5-10 years (OR 4.92, 95% CI 4.16-5.80 vs. OR 1.92, 95% CI 1.30-2.85/OR 2.14, 95% CI 1.49-3.09). Ben et al. conducted another similar study, published in 2011, compiling 35 cohort studies studying the association between DM and PC [61]. The findings showed that DM patients had a higher risk of PC (summary RRs = 1.94; 95% CI 1.66-2.27) and that this risk was independent of geographic location, sex, study design, alcohol use, BMI, and smoking status. Furthermore, there was an inverse connection between the relative risk of PC and the duration of DM, with the most significant risk of PC reported in individuals diagnosed during the first year [61]. Ekoé et al. conducted a study between 1984 and 1988 in Canada where they interviewed 179 cases of PC and 239 controls in a population-based case-control study (OR 2.52; CI 1.04-6.11) [62]. Results indicated that DM was almost three times as common in cases of PC (16%) than in controls (6%). In the study, 50% of PC patients had this illness before cancer, compared to 71% of the controls.

**Table 2:** Studies correlating DM and PC DM, diabetes mellitus; PC, pancreatic cancer; BMI, body mass index; RR, relative risk

Author	Design	No. of cases	No. of controls	Population/location	Conclusion
Zhang et al. (2019) [60]		7702	10,186	PC patients in China	The relationship between DM and PC had an odds ratio of 3.69. Individuals with DM for less than two years had a >2-fold increased risk of PC than those with DM for 2-4 or 5-10 years.
Aggarwal et al. (2013) [57]		100	100	PC patients of both sexes and a mean age of around 71.6 years	A greater prevalence of DM (68%) was found in patients with PC compared to age-matched patients with other cancers.
Elena et al. (2013) [59]	Case-control study	1621	1719	PC patients adjusted for multiple variables such as age, sex, etc. excluding patients with new-onset DM (<2 years)	DM increased the risk of PC by 40%. The risk was the highest for those with 2-8 years of DM. There was no connection for those with a DM duration of 9+ years.
Ben et al. (2011) [61]	35 cohort studies included in this meta-analysis				DM was associated with an increased PC risk in males and females (RRs = 1.94) independent of geographic locations, sex, study design, alcohol consumption, BMI, and smoking status.
Permert et al. (1993) [58]	Open study	44		Patients referred for radical operations for pancreatic carcinoma in Sweden	75% of PC patients had impaired glucose tolerance or diabetes.
Ekoé et al. (1992) [62]	Population-based case-control study	179	239	Patients with PC in Canada	DM was almost three times as common in cases of PC (16%) than in controls (6%).

Approach to this challenge

As observed above, obesity triggers several oncogenic pathways that help tumour cells survive, proliferate, and metastasise. Epidemiological evidence has also suggested weight loss can reduce PC mortality [63]. As a result, limiting the impact of obesity on cancer through methods such as weight loss, medical nutrition therapy, and bariatric surgery is vital for treatment [64]. One of the ways to achieve weight loss is through a hypocaloric diet consisting of ω3 polyunsaturated fatty acids (ω3 PUFAs), nuts, and olive oil, all of which lower the risk of obesity, metabolic syndrome, type 2 DM, PC, and overall cancer mortality [65-70]. It was also shown that physical exercise is more helpful in preventing weight gain than causing weight reduction. Thus, it is essential to incorporate it into daily activities [71]. Dietary adjustments with high fat and low carbohydrates, known as the ketogenic diets, have been effective. A study observed insulin withdrawal and considerable weight reduction in weeks in T2D patients [72]. Another study found that obese T2D patients on a low-carbohydrate (20 g/day) diet had lower plasma glucose and haemoglobin A1c levels and dramatically improved insulin sensitivity [73]. Higher protein diets with 1.2-1.6 g/kg/day of protein have been shown to cause increased satiety, weight loss, fat mass loss, and lean mass retention in adults. However, confirming a long-term protein effect is difficult because of the lack of dietary compliance [74].

While conservative treatment is the preferred and primary way of treatment, another targeted way of losing weight could be bariatric surgery. The most general bariatric operation in the United States was sleeve gastrectomy (SG; 49.4%) [75]. Research involving 1156 patients with severe obesity was divided into three groups: 418 patients who sought and received Roux-en-Y gastric bypass surgery, 417 patients who sought but did not receive surgery, and 321 patients who did not seek surgery. To determine type 2 DM, hypertension, and dyslipidemia, they performed clinical exams at baseline and 2, 6, and 12 years. After the Roux-en-Y gastric bypass, this study demonstrated long-term weight reduction and successful remission and prevention of T2D, hypertension, and dyslipidemia which could all, therefore, help in the development of PC ultimately [76]. In another trial, bariatric surgery improved insulin resistance, abnormal liver function tests, and liver histology. Weight reduction was also associated with a considerable rise in serum adiponectin levels after 6 and 12 months. In contrast, leptin levels declined, suggesting its anti-inflammatory nature and hence aid in arresting one of the possible mechanisms described in the development of PC [77].

Therapies targeted toward DM treatment

Another reasonable approach to target PC is to treat DM by lowering serum insulin. This involves taking blood glucose-lowering medications. Metformin, a commonly prescribed DM medication, improves insulin sensitivity in the liver by inhibiting gluconeogenesis and reducing blood glucose and insulin levels. Metformin inhibits cell oxidative phosphorylation, activating AMP-activated protein kinase (AMPK) [78]. Reduced ATP generation and cellular energy depletion result from this inhibition in cancer cells and reverse some of the proliferative pathways in obese people [79]. According to another research, metformin protects against carcinogen-induced PC in hamsters fed a high-fat diet [80]. Metformin disrupts the cell cycle, resulting in S phase arrest in PC. Metformin can interfere with specific receptors and affect the cell cycle since it lowers the oncoprotein level of epidermal growth factor receptors in PCCs. Kisfalvi et al. found that metformin inhibited insulin-induced PCC growth by disrupting the interaction between the insulin receptor and the G-protein coupled receptor [81,82]. While multiple types of research show that metformin has an anti-cancer effect, larger metformin dosages being utilised in mechanistic and animal studies, exceeding conventional therapeutic levels in people, call this into question [83].

Upcoming therapies targeting inflammation in the pathogenesis of PC

Recent advances in PC treatment include anti-fibrotic therapies that may target the fibrogenesis caused by PSCs. These include phytochemicals produced from medicinal plants or herbs that have a significant role in preventing or treating PC through various mechanisms. Some phytochemicals have exhibited anti-fibrotic effects against PSCs in recent years. The compounds in consideration are curcumin, resveratrol, rhein, emodin, green tea catechin derivatives, ellagic acid, embelin, eruberin A, and metformin. PSC migration and fibrogenesis were suppressed by specific phytochemicals such as curcumin that deactivated them by reducing their proliferation via the regulation of ERK1/2, P38 MAPK, and the SHH and PI3K/AKT signalling pathways. On the other hand, rhein and ellagic acid therapy deactivated PSCs by lowering the expression of fibrogenic markers (α-SMA) and soluble factors associated with pancreatic fibrosis, such as ECM (fibronectin and collagens) and TGF-β [84]. Another study investigated the effect of N-acetylcysteine (NAC) on PSCs and PCCs, and therefore, the microenvironment in PC. NAC decreased PSC viability, invasiveness, and migration at low concentrations, but not PCCs. In PSCs in a quiescent condition with numerous lipid droplets, NAC therapy significantly decreased oxidative stress and production of α-SMA and collagen type I. The study also indicated that the combination of NAC and pioglitazone inhibited tumour development and liver metastasis in vivo, with more minor stromal components and lower levels of oxidative stress [85].

Limitations

One of the potential mechanisms reviewed in this study is how obesity may lead to PC development through the development of DM. However, one study indicated no conclusive evidence on whether DM developed as a consequence of PC or vice versa, as many confounding factors, such as smoking, alcohol, and family history, may have affected the conclusion. Hence, further primary studies need to be conducted that adjust for any confounding factors to find a more precise link between obesity and PC. Additionally, this article focuses on obesity and DM as risk factors for PC development. While there are many mechanisms for PC development as described in this review, it is not easy to explore the precise mechanisms; hence, very few primary studies exist on the matter. Further research to study the mechanisms in depth is required for a more substantial evidence base.

## Conclusions

Based on this review study, and as discussed, we can infer that while there are other more substantial variables related to PC, such as smoking and family history, obesity is an emerging and robust competitor as one of the critical factors responsible for PC. The clinical significance of this review study is to establish a significant relationship between obesity and PC by delving into several mechanisms such as DM development, inflammation, beta-cell malfunction, and oxidative stress and discussing potential diagnostic techniques and, consequently, possible treatment approaches. Insulin resistance was one of the vital obesity-related pathways discussed here, and a vast body of literature supports this result. This review can help shape and innovate our future preventive and therapy methods for PC, especially for high-risk individuals such as those with a family history of PC and specific germline mutations. This can be done by emphasising its pathogenic processes and addressing these pathways by multiple methods, such as educating patients on weight-loss strategies and a healthy lifestyle, and targeting inflammatory pathways through the use of anti-inflammatory drugs and anti-fibrotic drugs that target the inflammatory, desmoplastic stroma associated with PC.

One of the topics covered in the evolution of PC through DM highlights how obesity might eventually lead to DM and, therefore, PC. This, however, may be a problem because PC has also been linked to the development of post-tumour DM. These issues can be tackled by including certain patients in trials who have a history of DM at least two years before PC development. Even though we provided evidence for the correlation between obesity and PC development, there has been the implication of the presence of certain confounding variables that could have affected the results. As a result, further studies are necessitated to reach a definitive conclusion that will aid in developing a better PC diagnosis, prevention, and treatment plan.
